# Value-Based Pricing and Reimbursement in Personalised Healthcare: Introduction to the Basic Health Economics

**DOI:** 10.3390/jpm7030010

**Published:** 2017-09-04

**Authors:** Louis P. Garrison, Adrian Towse

**Affiliations:** 1 Pharmaceutical Outcomes Research & Policy Program, Department of Pharmacy, Health Sciences Building, H375, 1959 NE Pacific St., H-375A, Box 357630, University of Washington, Seattle, WA 98195-7630, USA; 2 The Office of Health Economics, Southside, 7th Floor, 105 Victoria Street, London SW1E 6QT, UK; atowse@ohe.org

**Keywords:** personalized healthcare, personalized medicine, precision medicine, value-based pricing, value-based reimbursement, health economics, cost-effectiveness analysis, differential pricing, dynamic efficiency

## Abstract

‘Value-based’ outcomes, pricing, and reimbursement are widely discussed as health sector reforms these days. In this paper, we discuss their meaning and relationship in the context of personalized healthcare, defined as receipt of care conditional on the results of a biomarker-based diagnostic test. We address the question: “What kinds of pricing and reimbursement models should be applied in personalized healthcare?” The simple answer is that competing innovators and technology adopters should have incentives that promote long-term dynamic efficiency. We argue that—to meet this social objective of optimal innovation in personalized healthcare—payers, as agents of their plan participants, should aim to send clear signals to their suppliers about what they value. We begin by revisiting the concept of value from an economic perspective, and argue that a broader concept of value is needed in the context of personalized healthcare. We discuss the market for personalized healthcare and the interplay between price and reimbursement. We close by emphasizing the potential barrier posed by inflexible or cost-based reimbursement systems, especially for biomarker-based predictive tests, and how these personalized technologies have global public goods characteristics that require global value-based differential pricing to achieve dynamic efficiency in terms of the optimal rate of innovation and adoption.

## 1. Introduction

‘Value-based’ outcomes, pricing, and reimbursement are high on the list of buzzwords in discussions about health sector reforms these days. In this paper, we aim to discuss their meaning and relationship in the context of personalized healthcare. For purposes of addressing this question, we define ‘personalized healthcare’ as the receipt of care conditional on the results of a biomarker-based diagnostic test. We recognize that receipt of care can and should be conditioned on other characteristics of patients—such as age, gender, and preferences about health states—but we adopt this narrower definition for this discussion. For our purposes, personalized healthcare is synonymous with ‘personalized medicine’, ‘targeted treatment’, ‘precision medicine’, ‘individualized care’, and similar terms.

One can ask: “What kinds of pricing and reimbursement models should be applied in personalised healthcare?” The simple—but perhaps not very helpful—answer is that we want competing innovators and technology adopters to have incentives that promote long-term dynamic efficiency: that is, they would be participants—perhaps adversarial in some ways—in a health economic system that achieves the socially optimal rate of medical innovation in personalized healthcare. In 2004, Joseph Newhouse commented in an article about the US Medicare program that “the patent system is an effort to approximate a dynamically efficient price” [1]. This would apply to personalized healthcare as well.

We also need to clarify the difference between reimbursement and pricing. Reimbursement is about how and how much payers are willing to pay for covered services and products on behalf of their plan subscribers. Price is the amount that suppliers charge in order to provide a product or service to health plan members. We assume that suppliers are profit-maximizing firms that develop and sell medicines, complementary biomarker-based diagnostic tests, or both. However, they operate in a highly regulated environment in terms of development, manufacturing, and marketing—and, in some countries, in terms of reimbursement. National regulatory agencies monitor product quality and license use, and reimbursement authorities often negotiate prices or set them administratively. If suppliers want to charge prices higher than a payer is willing to accept, then either technologies (such as medicines and diagnostic tests) will not be available on the payer’s plan benefit package or patients will have to furnish a co-payment to make up the difference.

Our basic argument is that—to meet this social objective of optimal innovation in personalized healthcare—payers, as agents of their plan participants, should aim to send clear signals to their suppliers about what they value. They do this through their reimbursement practices and policies. We begin by revisiting the concept of value from an economic perspective, and summarize some of our previous work on how a broader concept of value is needed in the context of personalized healthcare. We next discuss the market for personalized healthcare and the interplay between price and reimbursement. We close by emphasizing the potential barrier posed by inflexible or cost-based reimbursement systems, especially for biomarker-based predictive tests, and how these personalized technologies have public goods characteristics that require global differential pricing to achieve dynamic efficiency in terms of the optimal rate of innovation.

## 2. What is Value? An Economic Perspective

From an economic perspective, the value of a good or service to an individual is what that individual would be willing to pay for it in monetary terms or give up in terms of other resources or time to receive it. This value also thus represents the ‘opportunity cost’ in that the individual has sacrificed the opportunity to use those resources in their next best alternative use. Of course, in market transactions, there may be a difference between what the individual would be willing to pay and the market price they face: that difference (if price is lower) is called the ‘consumer surplus’. If their willingness to pay is the ‘gross value’, then the consumer surplus could be considered the ‘net value’, i.e., gross benefit minus cost.

Despite President Trump’s claim that “nobody knew that healthcare could be so complicated”, economists and others have long recognized the complexities of the markets related to health and medical care. In his seminal article in 1963, Nobel-prize winning economist Kenneth Arrow pointed to “uncertainty” as the central feature that makes so many aspects of this market not only complicated, but also the source of what economists call ‘market failures’. For example, if consumers cannot be sure about the evidence as to the effectiveness of a medicine, they will be reluctant to use it even if it would be beneficial. Hence, national regulatory agencies, such as the European Medicines Agency (EMA) or Food and Drug Administration (FDA), certify the safety, quality, and efficacy of medicines. Arrow said: “...the special economic problems of medical care can be explained as adaptations to the existence of uncertainty in the incidence of disease and the efficacy of treatment” [2]. In other words, many of the institutional and regulatory features that we observe in medical care markets can be seen as remedies to correct these market failures.

Two of the biggest uncertainties facing individuals are whether they will fall ill and what to do about it if they do. Societies have created public and private health insurers (call them third-party ‘payers’) to allow individuals to pool these risks then to act as ‘agents’ on their behalf in the purchase or provision of medical care in the event of an illness. These payers also face uncertainties in acting on behalf of their subscribers (also called ‘enrollees’ or ‘plan participants’). They must decide what potential therapies are to be included in the ‘health benefit package’, and they may specify the details of which medicines, tests, or procedures are covered in the provision of specific therapies.

The fact that individuals seek—and most societies provide—health insurance to address these problems leads to another level of complications that makes value assessment challenging. Because of insurance protection, individuals no longer face the cost (i.e., technically, the full social marginal cost) of care at the point of consumption: hence, they have the incentive—called ‘moral hazard’—to over-use care. On the supply side, in systems with competing private insurers, the insurance providers have an incentive to enroll the lowest-cost patients while higher-risk patients are more likely to seek health insurance. This selection—either adverse or favorable depending on the point of view—can undermine insurance markets, as some are predicting for the US Affordable Care Act as a result of proposed reforms that might effectively end the requirement for people who regard themselves as healthy to buy insurance.

These market complexities also impact our ability to measure value in terms of willingness to pay since patients are often not directly spending health dollars at the point of care. In theory, researchers could do a ‘contingent valuation’ exercise and ask patients their willingness to pay an incremental insurance premium (or taxes) to cover an expensive procedure or medicine (e.g., a liver transplant or a curative hepatitis C drug) in the event they might need it in the future. However, the incremental amounts would typically be so small relative to the $10,000 per capita spent on average annually on medical care in the US [3], that individuals may not reliably or consistently be able to answer such a question. For example, about 1% of the US population has hepatitis C, but spreading that treatment cost over a life span of 80 years, even if we treated all of those with hepatitis C at a cost of $100,000 each, it would only amount to $12.50 on top of the $10,000 annual average. This is miniscule from an individual lifetime perspective. Of course, if we had to pay for it all in one year, it would be $100,000 × 2.3 million citizens or $230 billion—equivalent to the entire US prescription drug spending of $235 billion in 2015 [3]. So, from a short-term point of view, this creates a huge financing problem—which has generated a lot of discussion as well as caused health systems to ration access to these curative medicines.

The preceding presents an extreme case, but even here the incremental lifetime premium would be small relative to total health spending. Hence, health economists have developed an alternative approach—a ‘work-around’—to address this. We ask people how much they value a healthy year of life (called one quality-adjusted life year (QALY)), then we calculate—based on epidemiological and cost projections for treatment for a specific disease—what they should be willing to pay for it in the form of an incremental insurance premium.

This is implemented by comparing a new technology with the current Standard of Care (SoC) in terms of the ratio of the change in costs to the change in QALYs, which is called the ‘incremental cost-effectiveness ratio’ (*ICER*):

$$ICER=\frac{\mathrm{Cos}t\;\mathrm{of}\;\mathrm{new}\;\mathrm{technology}\;–\;\mathrm{Cos}t\;\mathrm{of}\;\mathrm{SoC}}{\mathrm{QALYS}\;\mathrm{generated}\;\mathrm{by}\;\mathrm{new}\;\mathrm{technology}\;–\;\mathrm{QALYs}\;\mathrm{under}\;\mathrm{SoC}}\;$$

A QALY can be thought of as life expectancy adjusted for quality of life (called ‘health-related’ utility) where each element of time is rated between 0 (death) and 1 (full or perfect health). This type of cost-effectiveness analysis using QALYs is often called ‘cost–utility analysis’.

If this ratio is less than the ceiling willingness-to-pay threshold, then it represents good value of money for the individual or health plan. In a US context, the ratio may vary from $50K/QALY up to $150K/QALY—or more depending on individual or disease. For the National Health Service (NHS) in the UK, the threshold used on behalf of the NHS is £20,000/QALY, ranging up to £50,000 for life-threatening conditions.

## 3. Expanded Cost-Effectiveness Analysis for Personalized Healthcare

Over the past 40 years, cost-effectiveness analysis using the QALY has proven to be a very useful tool for health sector decision-makers, though analysts and users are well aware of its limitations—both theoretical and practical. For example, the preference for quality of life vs. survival is unlikely to be constant over the lifetime of a patient, and assessing utility value for children or some cognitively impaired adults is challenging.

Personalized healthcare raises another set of issues around the use of this metric. The crux of the issue is that personalized treatment can create extra value for risk-averse individuals by reducing the uncertainty they face about response to treatment. Clearly, a risk-averse patient (or plan subscriber as a potential patient) would be willing to pay more for a treatment when they are more certain that they will benefit from it [4]. As has been noted in the literature, this can also create more value at an aggregate population level by improving adherence and uptake among responders, and helping the non-responders find their best alternative treatment more quickly. If we want to appropriately reward value creation to incentivize its production, then we need to consider all of these facets [5].

**Figure 1:**
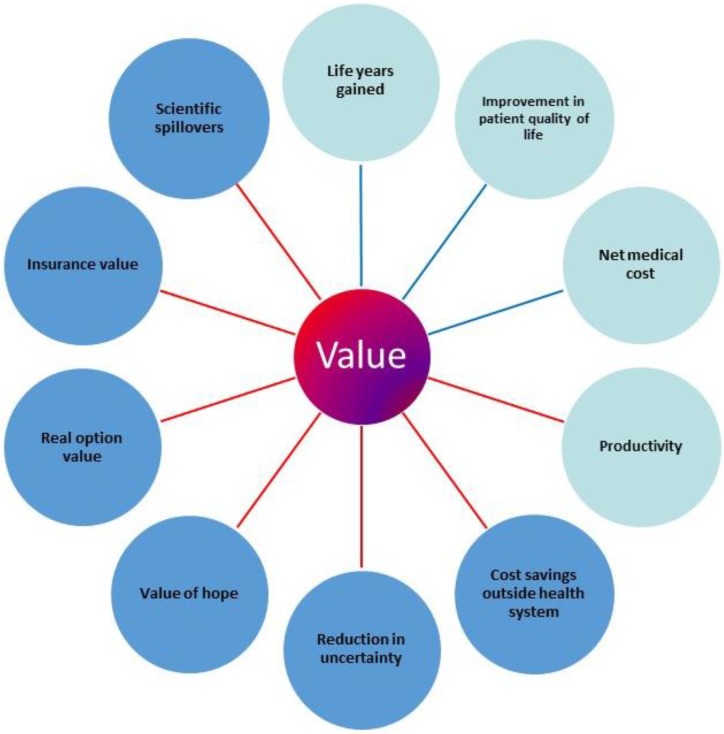
Dark blue circles: proposed information-related elements of value; Light blue circles: traditional Health Technology Assessment (HTA) and other societal elements of value; Blue line: value element in traditional HTA/health system perspective; Red line: additional value element also included in societal perspective. Source: Adapted from Figure 1 in Garrison et al. [6]

The recent literature has identified a number of additional elements of value, over and above life years gained and improvements in quality of life, potentially important to patients, but not captured in the QALY. These elements are depicted in Figure 1. We briefly describe them and their relevance to personalized healthcare.

If value is based on willingness to pay, one can ask what would a health plan member or patient be willing to pay for each of these elements as part of healthcare. The most obvious health-related elements are improvements in survival and in morbidity. The QALY aims to reflect these two elements. The other core element is the net medical cost impact: the cost of new intervention minus the cost of the standard of care. If the buyer can save $100 by avoiding an adverse event associated with the standard of care, they should be willing to pay up to $100 for that. These core elements are reflected in the *ICER*: they apply in personalized healthcare as well as in care that is not personalized. 

Perhaps the most significant next element, in general, is insurance value. Covering a new technology as part of a benefit package means that subscribers have some protection against the risk of catastrophic financial loss as well against risk of significant health loss [7,8]. However, in the case of personalized healthcare, a very important element is the reduction in uncertainty in the likelihood of benefit: non-responders can avoid the negative effects of adverse events and responders will gain a psychic benefit, viz., greater peace of mind, knowing that they are highly likely to benefit. This reduction in uncertainty has been called the “value of knowing” or “personal utility” [4,9]. “Real option value” is created when a treatment extends life so that the patient can benefit later in life from other new therapies that may currently be in the development pipeline [10]. The “value of hope” pertains to the idea that when survival prospects are poor, some patients may prefer to try interventions with a lower mean expected life extension than the comparator, but with a tail of survivors enjoying a substantial increase in life expectancy [11]. The element of “scientific spillovers” refers to what economists would call “knowledge externalities”, and they operate at a system-level [12]. A prime example is that the developers of personalized healthcare applications learn from the successes and failures of others in this field. So even failures in personalized healthcare applications can provide benefit from a health system or societal point of view.

With the exception of the reduction in uncertainty, none of these additional elements of value is specific to personalized medicine. However, they might all be relevant to the value created by a specific personalized healthcare application, and a full analysis will need to address this. Of course, these are still not all of the factors that a health plan’s HTA might want to consider. Other factors frequently mentioned include equity, severity of disease, unmet need, and end-of-life situations. Indeed, the recent US Second Panel on Cost-Effectiveness in Health and Medicine recommended an “Impact Inventory” that is even broader, considering impacts in other related sectors, such as education and the criminal justice system [13]. For further discussion of how an “extra-welfarist” perspective can also incorporate non-health elements of value in undertaking HTA in personalized medicine, see Rogowski et al. (2015) [14].

## 4. Pricing and Reimbursement in Personalized Healthcare to Reward Innovation

Because of the pervasiveness of health insurance, how healthcare markets use prices to mediate between demand and supply is different than in other markets. In most markets for goods and services, price adjusts to match (or ‘clear’) demand and supply: if the quantity demanded at a given price exceeds the quantity supplied, prices will rise, and if the quantity supplied is greater than the quantity demanded, prices will fall. However, healthcare markets—as Arrow indicated—are rife with uncertainty, informational asymmetry, and agency relationships. The payer—public or private insurer—is the agent for the plan members and negotiates prices and access to treatment. The physician works on the demand side as the agent/advisor for the patient, but also on supply side as a provider of treatments: the potential for conflict of interest, for example, by over-treating patients —labelled ‘supplier-induced demand’—is obvious. Legal and regulatory rules exist to counteract or limit this incentive.

As Professor Trump suggests, this is all very complicated. It is also dynamic: many health conditions are not curable and new ones arise, such as the Zika virus. There is a demand for innovation to address new diseases or conditions that are poorly managed, as well as to deal with age-related morbidity. All of this is not unique to personalized healthcare, but given the complexity of diseases, reflected in the heterogeneity of response of humans who differ genetically and in other ways, this push for innovation and improvement creates an incentive to better match the patient’s condition to the treatment. This is what personalized healthcare is all about.

It is well known that pricing and reimbursement practices (i.e., market-clearing mechanisms) that have developed in the healthcare sector vary across countries and types of health services. Five major medical inputs or suppliers in the ‘health production function’ of developed countries are: hospitals, physicians, medicines, devices, and diagnostic tests. Countries vary widely in the supply and use of these inputs, for example, hospital beds per capita and physicians per capita vary greatly among Organization for Economic Cooperation and Development (OECD) countries [15]. These inputs can be substitutes or complements in production, and hence their individual markets are inter-related: there will be ‘cross-price elasticities of demand’. As a substitute, a new, ‘high-price’ medicine may reduce the demand for physician services. As a complement, a new diagnostic that predicts responders to a medicine will make that medicine more valuable. 

Hospital services are paid for differently in different settings and countries. Some health systems rely on a top-down budgeting process while others pay per diem or per discharge. The diagnosis-related group (DRG) system developed in the US and adopted in many countries is a type of case-mix adjustment system that pays a fixed amount for patients falling into a particular illness category. Physicians are often paid on salary or via a fee for service per encounter or per procedure. In most developed countries, these systems are set up and administered through government. They are labelled ‘administered’ pricing and reimbursement systems. They aim, of course, to provide incentives for appropriate provision of services, and rewarding productivity, while maintaining cost control. However, in general, both hospital and physician reimbursement systems are more cost-based than value-based. [16]

Spending on hospital and physician care accounts for a majority of medical spending in most health systems, while prescription medicines are typically of the order of 10–15% [15]. The interactions among these inputs and their reimbursement is complex and variable. How prescription medicines are priced and reimbursed varies among health systems and countries. Manufacturers of innovative medicines are free to announce or propose ‘list’ prices in specific country markets, but then they negotiate with health systems, which may result in price discounts or rebates. Some countries—such as Australia and New Zealand—leverage their monopsony power in these negotiations. Countries often ‘reference’ or use the prices in other countries as part of their negotiations.

Innovative medicines, devices, and diagnostic tests differ from the hospital and physician care inputs in that the knowledge that they represent can be used all over the world—this knowledge is a ‘global public good’. It follows that everyone in the world has a stake in development and should be willing to support—to some degree—the underlying scientific research that generates them. While all innovations have the potential to meet the criteria to earn a patent, their ability to limit competition via that route varies: for example, there are many more follow-on compounds in some drug classes (e.g., statins for hypercholesterolemia) versus others (e.g., biologics for breast cancer).

While the demand for health improvements (‘endogenously’) pulls innovation to a large degree, it also seems likely that there is an (‘exogenous’) push in the march of science. The knowledge externalities cited above can yield breakthroughs at low marginal cost to society. The pull—in the case of innovative medicines—is the potential to obtain intellectual property (IP) rights and hence market power to behave as a monopolist or oligopolist for the remaining life of a patent, i.e., the portion (usually 8–12 years) of the 20 years that has not been used up in clinical development. Given this IP-protected power, manufacturers will charge what the market will bear, meaning they must anticipate—or argue for—the value that users will place on a new medicine. In the case of personalized healthcare, given the variability in human biology and genetics and the complexity of disease, only a subset of treated patients may be true ‘responders’. Since the benefit–risk balance will be more favorable in such a subset, the value will be greater, and a higher price could be justified.

Since the sequencing of the genome early this century, there has been tremendous interest and enthusiasm for the idea that that genetic information could be used to target therapies to the ‘right’ patient, but progress has been much slower than many expected [17]. These predictive tests have been called “complementary diagnostics” as they are economic ‘complements’ to be used in conjunction with medicines [12]. The biggest reason for this slow progress is probably that science has proven to be more difficult than expected [18]. Genomic markers often have limited ability to predict response to treatment in complex diseases. Even the expansion of the range of biomarkers to include, for example, proteomic and metabolomic biomarkers has not produced rapid progress. Still, there is great promise and considerable investment is being made.

Over the past 30 years, one important economic reality shaping drug development has been the pervasive influence of the US marketplace given its predominance as the most profitable market. This affects the therapeutic area selection as well as clinical development program strategy, including trial design. In the US, manufacturers of new medicines are free to set their prices, and reimbursement and coverage are negotiated with payers. On the other hand, pricing and reimbursement for diagnostics in the US has been a different matter historically as reimbursement has operated via an administered, cost-based system. The reimbursed amounts were sometimes derived by “cross-walking” to the costs of similar tests or “code stacking” by adding the costs of the separate steps in processing the sample [19]. The contribution of the diagnostic to the ‘value’ as defined above was not considered. So, if there would be extra value in the combination of a complementary test-medicine pair, the medicine would have a great advantage to capture that value. All of this means that manufacturers of drugs and tests have less incentive to develop a test, especially after the drug is already on the market. Drug manufacturers could—and reportedly do, increasingly—consider developing a test in parallel with drug, but given the high rate of failure in drug development itself, adding this parallel development track adds scientific complexity and further risk and cost. Furthermore, if it delays the drug development—resulting in lost patent life—then it can be very costly to the bottom line. The lack of value-based reimbursement for tests is a barrier for diagnostic manufacturers to develop tests after the product is on the market. If they cannot raise the money for the validation trials, then the ‘science’ part cannot proceed.

The argument has been made for flexible, value-based reimbursement for the medicine and the complementary diagnostic to reduce the impact of this barrier [20,21]. Furthermore, it has been pointed that the division of value between economic complements is fundamentally arbitrary. The value of a peanut butter-and-jelly sandwich is in the combination, the costs of the three inputs can be found out, but each is essential to the total value. However, in the case of a test-drug combination, where rewards to IP can be involved, there is the concern about dynamic efficiency emphasized above. One proposal is to split the total value by (a) rewarding the medicine developer for the net health gain and cost-offsets in the responder target population, and (b) rewarding the diagnostic manufacturer for avoiding health losses and costs associated with adverse events in the non-responder group, which presumably derives little or no clinical efficacy. As mentioned above, the test-drug combination also provides value by reducing uncertainty for patients by creating value (of knowing) for patients who are risk averse to uncertainty.

## 5. Pricing, Reimbursement, and Sustainability with Personalized Healthcare

The developers of innovative pharmaceuticals, devices, and diagnostic tests face a complex global marketplace. First, since their products have global public goods attributes, many countries can come into play, and each has its own health delivery system, priorities, and approach to pricing and reimbursement. Still, the benefits of succeeding in the US market are apparent and must be balanced with increasing interest and income growth in the rest of the world—developed and developing.

For the innovative pharmaceutical industry to be sustainable and robust, the revenues generated through the pricing and reimbursement system must at least be on a par with the growth of R&D costs. Broadly speaking, overall industry productivity in terms of the number of new compounds has been fairly flat over the past 11 years—averaging 30–35 new compounds per years. However, real R&D costs have been rising at 8.5% per annum and the latest estimate of the average cost per new molecule entity is $2.6 billion [22]. Reflecting the same trends, the average returns have been falling to nearly zero for the latest cohort that could be analyzed [23]. For the industry to be sustained with the same number of firms, average price (e.g., per unit of health gain) must rise, and there is some evidence of this [24]. Not surprisingly, the increasing concerns of the public about rising drug prices are in the news every day.

What is or what will be the impact of more personalized healthcare on these trends? The answer to that is not clear at this stage. If R&D costs continue to rise, then medicines targeted to smaller indications will require higher prices. Trusheim and Berndt (2010) have argued that small populations will not get new drugs unless prices are much higher [25]. Danzon and Towse (2002) argued that personalized medicine would typically involve targeting interventions at patients who respond [26]. Health gain would be concentrated in these sub-populations, rather than spread across a broader patient group including responders and non-responders. As a result, the same health gain would be concentrated in a smaller patient group, justifying a higher price at any given cost-per-QALY threshold. This will offset the declining market size to a degree. For rare conditions involving catastrophic health loss, higher prices for any given QALY gain can also be justified, in part by the greater ‘insurance value’ provided—one of the novel elements cited above: Jena and Lakdawalla (2017) make the case that rare diseases also usually involve very substantial health loss with spillover effects to caregivers—usually other family members [27]. Most citizens would pay a premium over and above the expected QALY value to be in a payer system that provided drugs that could prevent or reverse that health loss.

## 6. Issues in Global Financing: The Need for Global Differential Pricing

Innovative technologies whether drugs or their complementary diagnostics can potentially benefit all 7.4 billion inhabitants of Earth, and as such, have global public good characteristics. Economists have long argued with such economic goods, the optimal R&D financing system would be based on global differential pricing across countries based on ability to pay (income) and willingness to pay for improved health [28]. This would apply in personalized healthcare as well.

Danzon et al. (2015) have argued that if each payer uses a ceiling willingness-to-pay threshold that reflects the willingness and ability of their enrollees to pay for health gain, and limits maximum reimbursement for drugs and diagnostics to this amount, then, globally, the correct signals will be given to companies to invest in R&D [29]. The benefits of taking a global view are well illustrated by the experience with vaccines for infectious diseases and with treatments for HIV. In both of these cases, there has been both global access and differential pricing across jurisdictions.

## 7. Conclusions: A Global Imperative Needs Global Financing 

The unique nature of innovative medicines and their complementary diagnostics as economic goods clearly implies that a global perspective is needed on their development and financing. Pricing and reimbursement should be value-based, but also flexible, given that value changes over time and varies by jurisdiction. All of this is not to say that we should be spending more or less on medicines or on personalized healthcare. Our point is about dynamic efficiency and global public goods: we will not get the optimal amount of spending on personalized healthcare unless we can design and implement global pricing and reimbursement systems that can support this aim with appropriate incentives. Producing the science and evidence to support personalized healthcare is costly, and if pricing and reimbursement policies within and across countries and between medicine and diagnostic development do not efficiently share these costs and reward value appropriately, then the global rate of innovation will be sub-optimal with a long-run adverse impact on population health.
